# Sensitivity and specificity of the new Bio-Rad HIV screening test, Access HIV combo V2

**DOI:** 10.1128/jcm.00095-24

**Published:** 2024-03-27

**Authors:** Vincent Guiraud, Yann Ciczora, Muriel Cardona, Christine Defer, Sandrine Gréaume, David Nogues, Agnès Gautheret-Dejean

**Affiliations:** 1 AP-HP, Hôpitaux Universitaires La Pitié Salpêtrière-Charles Foix, Service de Virologie, Paris, France; 2 Bio-Rad Laboratories, Steenvoorde, France; 3 Bio-Rad Laboratories, Marnes-La-Coquette, France; 4 Etablissement Français du Sang (EFS) Hauts de France—Normandie, Lille, France; 5 Etablissement Français du Sang (EFS) Hauts de France—Normandie, Bois-Guillaume, France; 6 Université Paris cité, INSERM UMR-S 1139 Physiopathologie et pharmacotoxicologie placentaire humaine: microbiote pré & post-natal, Paris, France; St. Jude Children's Research Hospital, Memphis, Tennessee, USA

**Keywords:** Access HIV combo V2, HIV, sensitivity, specificity, accuracy, serology

## Abstract

Diagnosing of human immunodeficiency virus (HIV) types 1 and 2 requires a screening with a highly sensitive and specific enzyme immunoassay and a low detection limit for the HIV-1 p24 antigen to minimize the diagnostic window. The objective of the study was to determine the sensitivity, specificity, and p24 limit of detection of the Access HIV combo V2 assay. Retrospective part of sensitivity: 452 HIV-1 positive samples from 403 chronic (9 different HIV-1 group M subtypes, 22 different HIV-1 group M CRFs, and 3 HIV-1 group O), 49 primary HIV-1 infections, 103 HIV-2 positive samples assessed at Pitié-Salpêtrière Hospital, 600 untyped HIV-1, 10 subtype-D, and 159 untyped HIV-2 samples assessed in Bio-Rad Laboratories. Prospective part of clinical specificity: all consecutive samples in two blood donor facilities and Pitié-Salpêtrière (6,570 patients) tested with Access HIV combo V2 and respectively Prism HIV O Plus (Abbott) or Architect HIV Ag/Ab Combo (Abbott) for Ag/Ab screening, and Procleix Ultrio (Gen Probe) for HIV RNA screening. Limit of detection for p24 antigen was assessed on recombinant virus-like particles (10 HIV-1 group M subtypes/CRFs, HIV-1 group O). Sensitivity [95% confidence interval (CI)] of Access HIV combo V2 was 100% (99.63–100) for HIV-1 chronic infection, 100% (98.55–100) for HIV-2 chronic infection, and 100% (93.00–100) for HIV-1 primary infection. Specificity (95% CI) was 99.98 (99.91–100). Limit of detection for p24 antigen was around 0.43 IU/mL [interquartile range (0.38–0.56)], and consistent across the 11 analyzed subtypes/CRFs. Hence, with both high sensitivity and specificity, Access HIV combo V2 is a suitable screening assay for HIV-1/2 infection.

**IMPORTANCE:**

Bio-Rad is one of the leading human immunodeficiency virus (HIV) screening test manufacturers. This laboratory released in 2021 their new version of the Access combo HIV test. However, to date, there have been no studies regarding its performance, especially its limit of detection of the diverse p24 antigen. We present the sensitivity (chronic and primary HIV-1 infection and HIV-2 chronic infection), specificity (blood donors and hospitalized patients), and raw data for the p24/seroconversion panels the manufacturer gave to the European agencies.

## INTRODUCTION

The first target to achieve the 2025 World Health Organization target of ending the human immunodeficiency virus (HIV) pandemic is that at least 95% of people living with HIV know their status (1). HIV diagnosis faces three main challenges. Firstly, it has to detect antibodies directed to a remarkable range of diverse antigens from both HIV-1 and HIV-2 (2, 3). Secondly, as a non-negligible part of HIV transmission occurs early after an infection (4–6), diagnostic window should be as reduced as possible. As a consequence, the assay must detect the HIV-1 specific p24 antigen at the lowest limit of detection possible. Lastly, as a false positive HIV diagnosis can have deleterious consequences, a high specificity is needed (7–9).

HIV diagnosis tests have remarkably improved since the beginning of the HIV pandemic, with current recommended ones, or fourth generation assays, able to detect with high sensitivity and specificity all circulating variants with a diagnostic window of about 2 weeks after infection (1, 10–12).

The objective of this study was to assess the sensitivity and specificity of Bio-Rad’s new fourth generation HIV test, Access HIV combo V2 on both real-life settings and well-characterized commercial panels. Additionally, we aimed to establish its limit of detection for the p24 antigen using diverse, well-characterized commercial panels.

## MATERIALS AND METHODS

### Study design—sample collection

This study included a multicenter retrospective part of clinical sensitivity and a multicenter retrospective and prospective part of clinical specificity.

Two sites were involved in clinical sensitivity. The Service of Virology at the Pitié-Salpêtrière Hospital (Paris, France) provided 452 HIV-1 positive samples from 403 chronic and 49 primary HIV-1 infected patients and 103 HIV-2 positive samples from chronically infected patients. HIV-1 serum samples from chronically infected patients were selected to account for a large part of HIV-1 diversity (Table S1): 9 different HIV-1 group M subtypes, 22 different HIV-1 group M CRFs, and 3 HIV-1 group O. Serum samples from HIV-1 primary infection, of which nine were positive for p24 antigen only [Stages II and III (13)], were mostly of HIV-1 subtype B, CRF02 and CRF06 (Table S1). HIV-antibody and p24 positivity were assessed using Architect HIV Ag/Ab Combo (Abbott, Rungis, France) and Liaison XL HIV Ab/Ag (Diasorin, Antony, France) as screening assays with New LAV Blot I and II (Bio-Rad Laboratories, Marnes-la-Coquette, France) as confirmatory and differentiation assays. Subtypes and recombinant forms of HIV-1 strains were determined using molecular assay as previously described (14). Samples were stored frozen at −20°C until use. HIV-positive samples tested at the Bio-Rad Laboratories originated from commercial panels supplied by Seracare, Zeptometrix, and Biomex for seroconversion panels, as detailed in Table S2. Days to first reactive results were compared with the Architect assay. For the Architect assay, we used seroconversion panel data provided by the manufacturer, followed if unavailable by the FDA’s published data. If these data were still missing, we extended our review to previously published studies. Chronic HIV samples tested in Bio-Rad Laboratories included a total of 600 ungenotyped HIV-1, 10 HIV-1 subtype D samples, and 159 ungenotyped HIV-2 samples.

For the retrospective part of clinical specificity, 203 frozen serum samples from HIV-negative pregnant women who consulted at Pitié-Salpêtrière Hospital from April 2019 to January 2020 and 10 HTLV+/HIV− serum samples were analyzed. HIV status was assessed using Architect HIV Ag/Ab Combo.

The prospective part of clinical specificity included the following: fresh serum or plasma samples from all consecutive patients with an HIV testing at Pitié-Salpêtrière Hospital from 30 January 2020 to 17 March 2020 (1,509 samples), all samples from consecutive blood donors at the Etablissement Français du Sang (EFS) of Bois-Guillaume (France) from 28 January 2020 to 11 February 2020 (2,512 samples), and all consecutive blood donors from the EFS of Lille (France) from 10 February 2020 to 13 March 2020 (2,549 samples).

### Access HIV combo V2 assay

The Access HIV combo V2 assay is a paramagnetic particle, semi-quantitative chemiluminescent immunoassay designed to detect HIV-1 p24 and HIV-2 p26 antigens, and antibodies to HIV-1 and HIV-2 in human serum or plasma. The test is configured to run on Beckman Coulter’s Access immunoassay systems with a run time of 30 minutes. Results are expressed as S/CO ratio with S/CO < 0.9 considered as non-reactive, S/CO ≥ 1 reactive, and S/CO [0.9 to <1] gray zone. This test does not distinguish antigen from antibody reactivity.

### Sample processing

For the retrospective parts of sensitivity and specificity, all samples were tested with the Access HIV combo V2 according to the manufacturer’s recommendations. Tests found negative for the study of sensitivity were planned to be repeated once.

For the prospective part of specificity, all samples were tested in parallel with Access HIV combo V2 and either Architect HIV Ag/Ab Combo at the Pitié-Salpêtrière Hospital or Prism HIV O Plus (Abbott) and Procleix Ultrio (Novartis Diagnostics, Emeryville, CA, USA) at the EFS centers. All gray zone and reactive samples were retested in duplicate, followed, if remaining gray zone or reactive, by a confirmatory assay. Confirmatory assays were conducted using either New LAV Blot I and II at Pitié Salpêtrière Hospital or INNO-LIA HIV I/II Score (Innogenetics, Gent, Belgium) at the EFS centers.

### p24 and p26 antigens analytical sensitivity

Access HIV combo V2 assay sensitivity for p24 and p26 antigens was assessed on the Access 2 platforms using the NIBSC/WHO p24 antigen standards (NIBSC 90/636 and 16/210) and p26 antigen (NIBSC 16/236) with the following dilutions: 1:2, 1:4, 1:8, 1:16, and 1:32. Both antigens were diluted in sterile water.

### Statistical analysis

Statistical analysis was conducted using R version 4.2.1 software (15). Categorical variables were expressed as numbers (percentages) and continuous variables as medians [interquartile range (IQR)]. The 95% confidence intervals (95% CIs) were calculated using Wilson’s confidence interval for proportions (16). The limit of detection was assessed for p24 antigen using linear regression to calculate the amount of p24 detected for an S/CO ratio of 1. Comparison was done using *χ*^2^ test for categorical variables with a significance assigned at a *P* value <0.05. Sample size was determined according to the European Commission’s decision on technical specifications for *in vitro* diagnostic medical devices (17).

### Role of the study sponsor

The sponsor provided reagents and automated equipment used in this study and was responsible for data collection. First and last authors had full access to the study database, generated statistical analyses, prepared the first draft of the manuscript, and made the decision to submit the manuscript for publication. Y.C., D.N., and M.C. are employed by Bio-Rad. V.G., A.G.D., C.D., and S.G. received no personal funding from the study sponsor.

## RESULTS

### Sensitivity on chronic HIV infection

All the 403 HIV-1 samples from the retrospective study collected at the Pitié-Salpêtrière Hospital as well as the 25 HIV-1 samples collected during the prospective study of specificity were reactive, yielding a sensitivity of 100% [95% CI (99.11–100)]. Each of the 600 ungenotyped and 10 HIV-1 subtype D samples tested in Bio-Rad Laboratories were also reactive, owing a 100% [95% CI (99.37–100)] sensitivity in Bio-Rad Laboratories.

All the 103 retrospective chronically HIV-2 samples collected at Pitié-Salpêtrière Hospital as well as all the 159 HIV-2 samples at Bio-Rad Laboratories were positive, owing a sensitivity of 100% [95% CI (96.40–100)] at Pitié-Salpêtrière and 100% [95% CI (97.64–100)] in Bio-Rad Laboratories.

**Fig 1:**
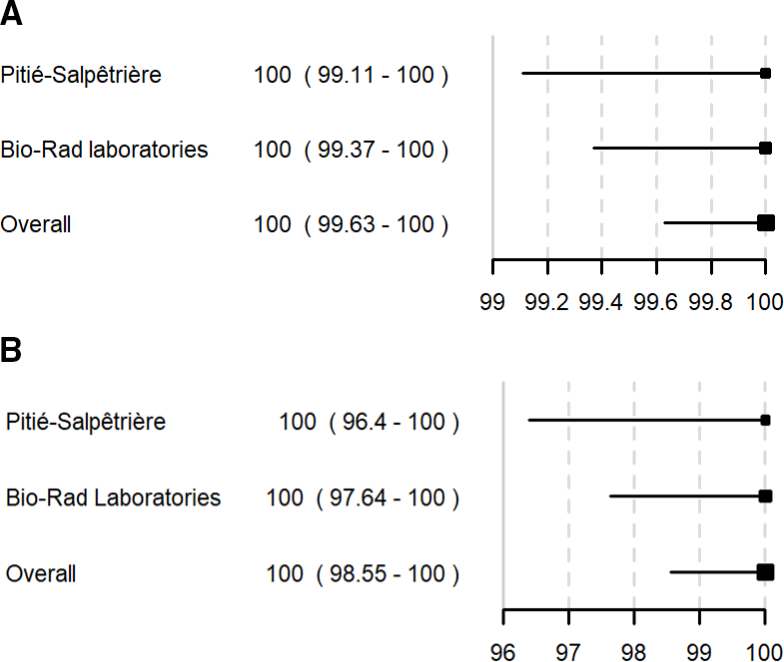
Sensitivity of the Access HIV combo V2 for HIV-1 (A) and HIV-2 (B) chronic infection.

Pooled estimated sensitivity for the diagnosis of chronic HIV-1 infection was 100% [95% CI (99.63–100)] and for the diagnosis of chronic HIV-2 infection at 100% [95% CI (98.55–100)]. These results are summarized in Fig. 1, and S/CO values are reported in Fig. S1.

### Sensitivity for HIV-1 primary infection

All the 49 retrospective and 2 prospective samples from primary HIV-1 infection collected at Pitié-Salpêtrière Hospital were found reactive on Access HIV combo V2, yielding a sensitivity of 100% [95% CI (93.00–100)]. Corresponding S/CO values are reported in Fig. S1.

A total of 415 samples from 41 commercial seroconversion panels were tested in Bio-Rad Laboratories. Results aligned closely with Architect (reference assay). Day to first reactive result was identical for 35 (85.4%) of them, while Access outperformed for 5 (12.2% with, respectively, 5, 6, 7, 3, and 5 days later for the Architect) and underperformed for 1 (2.4% with 4 days earlier for the Architect) of them (Table S2). Discrepant results between the two assays are summarized in Table 1.

**TABLE 1:** Commercial seroconversion panels with conflicting results between Access HIV combo V2 and Abbott’s Architect

Vendor	Sample ID	Days to first reactive result		Difference of first reactive result (days)^*a*^	Source for Architect results
		Access Combo V2	Architect		
Seracare/BBI	PRB944	2	7	5	FDA notice
	PRB945	7	13	6	Sano et al. (18)
	PRB953	7	3	−4	Manufacturer
	PRB957	16	23	7	FDA notice
	SC9018	25	28	3	Manufacturer
	SC12008	23	28	5	Manufacturer

^*a*^ A positive number indicates that Access Combo V2 is reactive before Architect, while a negative result indicates that Architect is reactive before Access Combo V2.

### Specificity

Blood donor samples, routinely tested at EFS Hauts de France—Normandie in Lille (*n* = 2,549) and Bois-Guillaume (*n* = 2,512) with Prism HIV O Plus and Procleix Ultrio were prospectively analyzed in parallel with Access HIV combo V2. No initial false-reactive sample was identified at the first site, while at the second site, four samples were found initial false reactive (IR). These four IR samples were negative after repeating in duplicate. This resulted in an overall IR specificity for blood donors of 99.92% [95% CI (99.80–99.97)] and an overall specificity after repeat testing of 100% [95% CI (99.92–100)]. In comparison, Prism HIV O Plus assay gave four repeated reactive (RR) false-reactive samples on the blood donor’s population. There was no false-reactive sample identified with the Nucleic Acid Amplification Test (NAAT) Procleix Ultrio, owing a specificity of 100% [95% CI (99.92–100)]. Of note, no sample was identified as true reactive.

At Pitié-Salpêtrière Hospital, among the 1,509 samples tested prospectively in parallel with Architect, 27 (1.8%) were found to be true reactive samples (25 chronic and 2 primary infections), while 5 were IR and 1 was repeatedly false reactive. As so, the hospitalized patient IR specificity assessed was 99.66% [95% CI (99.74–99.83)] and RR specificity was 99.93% [95% CI (99.62–99.99)]. Architect specificity was identical with two other repeatedly false-reactive samples. We also performed a retrospective exploratory analysis on 203 hospitalized HIV-negative pregnant women. There was no false-reactive sample, owing a specificity of 100% [95% CI (98.1–100)]. Of note, RR hospitalized patient specificity was not statistically significantly lower than blood donor specificity (*P* = 0.51). Also, we performed an exploratory analysis on 10 HTLV-1 positive/HIV-negative samples. One sample tested reactive with an S/CO ratio of 1.46, repeated at 1.86 and 1.81. Architect was also reactive on this sample. As the HIV Western blots were negative, this sample was considered as a false-reactive sample.

**Fig 2:**
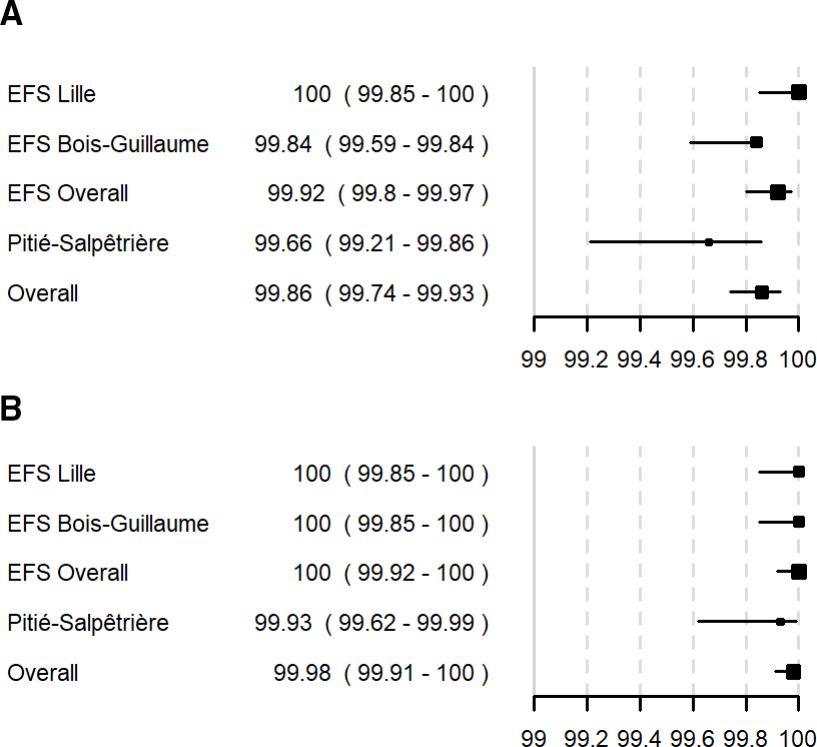
Specificity of the Access HIV combo V2 on first result (A) and after repeat (B).

As so, pooled (blood donors and hospitalized patients) IR specificity was 99.86% [95% CI (99.74–99.83)], and RR specificity was 99.98% [95% CI (99.91–100)]. Specificity results are summarized in Fig. 2, and S/CO distribution for negative results is summarized in Fig. S1.

### Analytical p24 and p26 antigen sensitivity

The limits of detection for p24 and p26 were assessed using WHO standardized panels (Table 2). The first international reference sample for HIV-1 subtype B had a detection limit of 0.39 IU/mL. The limit of detection for the p24 antigen was consistent across the 11 HIV-1 subtypes, ranging from 0.27 IU/mL to 0.58 IU/mL with a median of 0.43 IU/mL [IQR (0.38–0.56)]. Since p26 had no assigned unitage, its limit of detection was determined using serial dilution with samples yielding positive results up to a dilution of 1/8.

**TABLE 2:** Analytical sensitivity of the Access HIV combo V2 assay on the Access platform for HIV-1 p24 antigen according to group, subtypes, and CRFs

HIV-1 subtype	Analytical sensitivity (IU/mL)
B^*a*^	0.39
A1^*b*^	0.56
B^*b*^ (16/214)	0.27
B^*b*^ (16/216)	0.35
C^*b*^	0.47
D^*b*^	0.53
F1/CRF12_BF/BFrec^*b*^	0.43
G^*b*^	0.56
CRF20_BG^*b*^	0.38
CRF01_AE^*b*^	0.56
CRF02_AG^*b*^	0.36
H^*b*^	0.42
Group O^*b*^	0.58

^*a*^ WHO reference panel 90/636.

^*b*^ WHO reference panel 16/210.

## DISCUSSION

Overall, Access HIV combo V2 displayed high sensitivity for both chronic HIV-1 samples with a sensitivity of 100% [95% CI (99.63–100)] and for HIV-1 primary infection samples with a sensitivity of 100% [95% CI (93.00–100)]. The sensitivity for HIV-2 infection was also 100% [95% CI (98.55–100)]. Specificity was also high, at 99.98% [95% CI (99.91–100)]. Limit of detection of p24 antigen was low, around 0.43 IU/mL, and consistent across the analyzed HIV-1 groups, subtypes/CRFs.

27, 28

High sensitivity and specificity were expected findings, consistent with previous reports from all other commercial fourth generation assays (11, 18–23). Analytical sensitivity for p24 antigen was around 0.43 IU/mL, consistent across the 11 HIV-1 subtypes/CRFs. This finding contrasts with the previous Access HIV combo version, which had a limit of detection for subtype B around three times higher (24) and performed very poorly on non-subtype B samples with limit of detection often over 10 IU/mL (25). Compared with published data (26), Access HIV combo V2 [median (IQR) p24 Ag limit of detection of 0.43 IU/mL (0.38–0.56 IU/mL)] had a similar limit of detection as Architect HIV Ag/Ab Combo [0.57 IU/mL (0.43–0.64 IU/mL)] and BioPlex 2200 HIV Ag-Ab assays [0.27 IU/mL (0.21–0.36 IU/mL)], outperformed Liaison XL Murex HIV ab/Ag and Elecsys HIV combi PT assays [0.67 IU/mL (0.58–0.72 IU/mL)], but underperformed if compared with the Elecsys HIV Duo assay [0.33 IU/mL (0.30–0.37 IU/mL)], as described in Table S3. However, we were unable to link these gaps in detection thresholds to potential differences in HIV-1 window period, as to date, we have not managed to gather any non-reactive fourth generation HIV-1 sample that was positive on HIV-1 NAAT. As this contrasts with current guidelines that advise the use of NAAT in this setting (27, 28), further studies are needed to address the relevance of this guideline in the setting of increasing p24 sensitivities of fourth generation assays.

This study has several limitations. The genotypes of HIV-1 responsible for primary infection at Pitié-Salpêtrière were predominantly HIV-1 group M subtype B and CRF02_AG, reflecting the French and European epidemiologies (2). Furthermore, since their sera were primarily screened using the Architect platform, this part of the study could not ascertain if Access had a shorter window period. Regarding the commercial seroconversion panel, a similar bias toward subtype B might exist since most blood samples originate from US patients. Furthermore, Architect’s results were extracted from the manufacturer’s data or previously published studies (Table S2) instead of being generated from samples stored within the same conditions. The limits of detection for p24 antigen from different HIV-1 subtypes and p26 antigen were derived from commercial recombinant virus-like particles rather than patient sera, to facilitate future comparison. This specification, however, represents a surrogate marker for HIV-1 primary infection and cannot be rigorously translated into window periods. Finally, this assay is designed to detect p26 antigen, to shorten the window period for HIV-2 infection. However, due to a lack of HIV-2 primary infection samples, we were unable to validate this hypothesis.

As a conclusion, Access HIV combo V2, with both high sensitivity and specificity, is a suitable screening assay for HIV-1 and HIV-2 infections.

## Data Availability

Raw data regarding p24 and p26 limit of detection are included as supplemental material. S/CO values of this study are available on request from the corresponding author.

## SUPPLEMENTAL MATERIAL

Raw data jcm.00095-24-s0001.xlsx Raw data for p24 and p26 limit of detection and for seroconversion panels.

Fig S1A jcm.00095-24-s0002.tiff Image of Fig. S1A.

Fig S1B jcm.00095-24-s0003.tiff Image of Fig. S1B.

Figure S1 jcm.00095-24-s0004.docx S/CO values for HIV-positive samples (A) and HIV-negative samples (B), according to the sites.

Supplemental tables jcm.00095-24-s0005.docx Tables S1 to S3.

ASM does not own the copyrights to Supplemental Material that may be linked to, or accessed through, an article. The authors have granted ASM a non-exclusive, world-wide license to publish the Supplemental Material files. Please contact the corresponding author directly for reuse.
