# Supplementary figures and images for: Sensitivity and specificity of the new Bio-Rad HIV screening test, Access HIV combo V2
Source: J Clin Microbiol. 2024 Mar 27;62(5):e00095-24. doi: 10.1128/jcm.00095-24 (PMC11077987; doi:10.1128/jcm.00095-24)

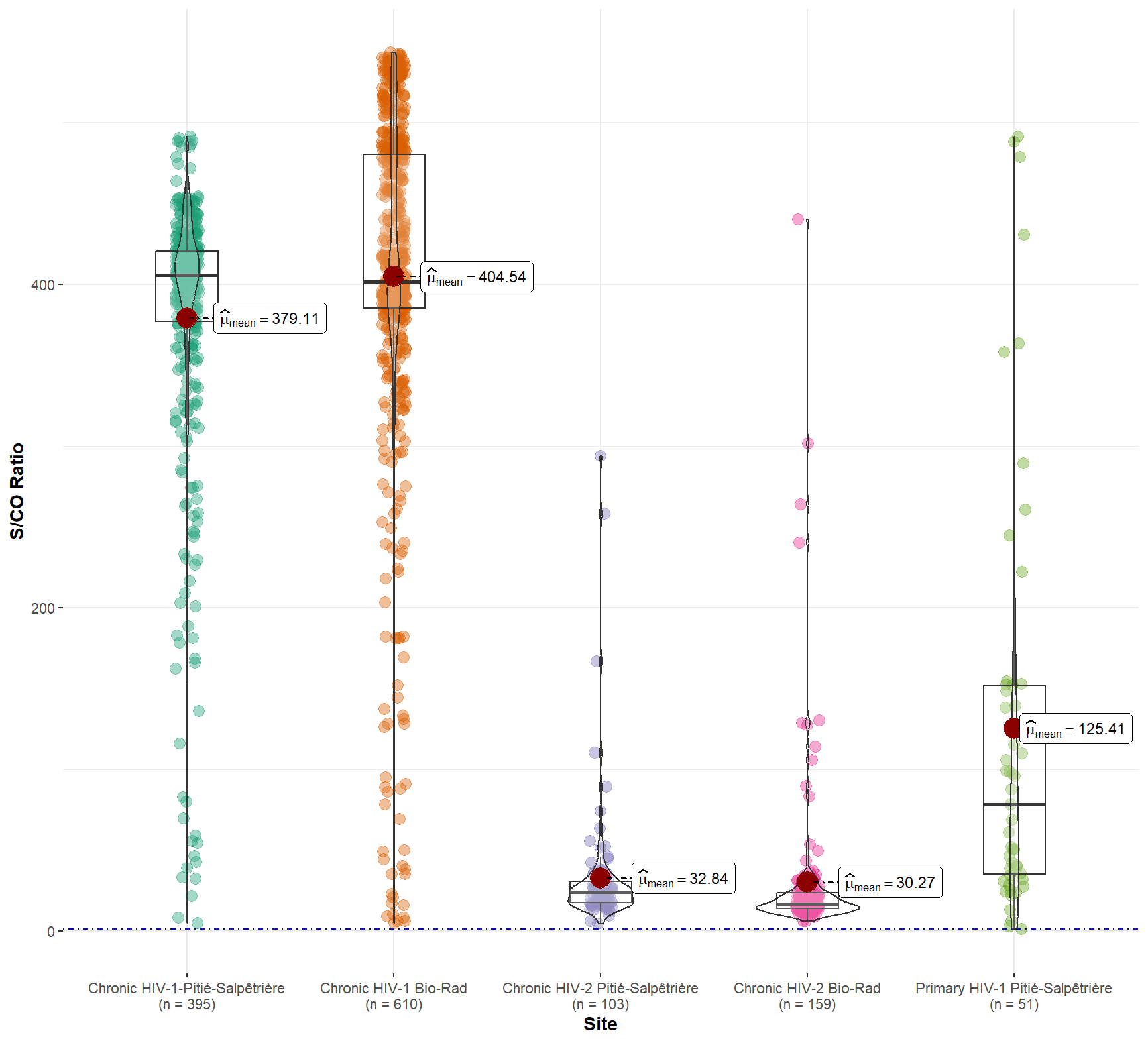

Supplement: Fig S1A — Image of Fig. S1A. [file jcm.00095-24-s0002.tiff]

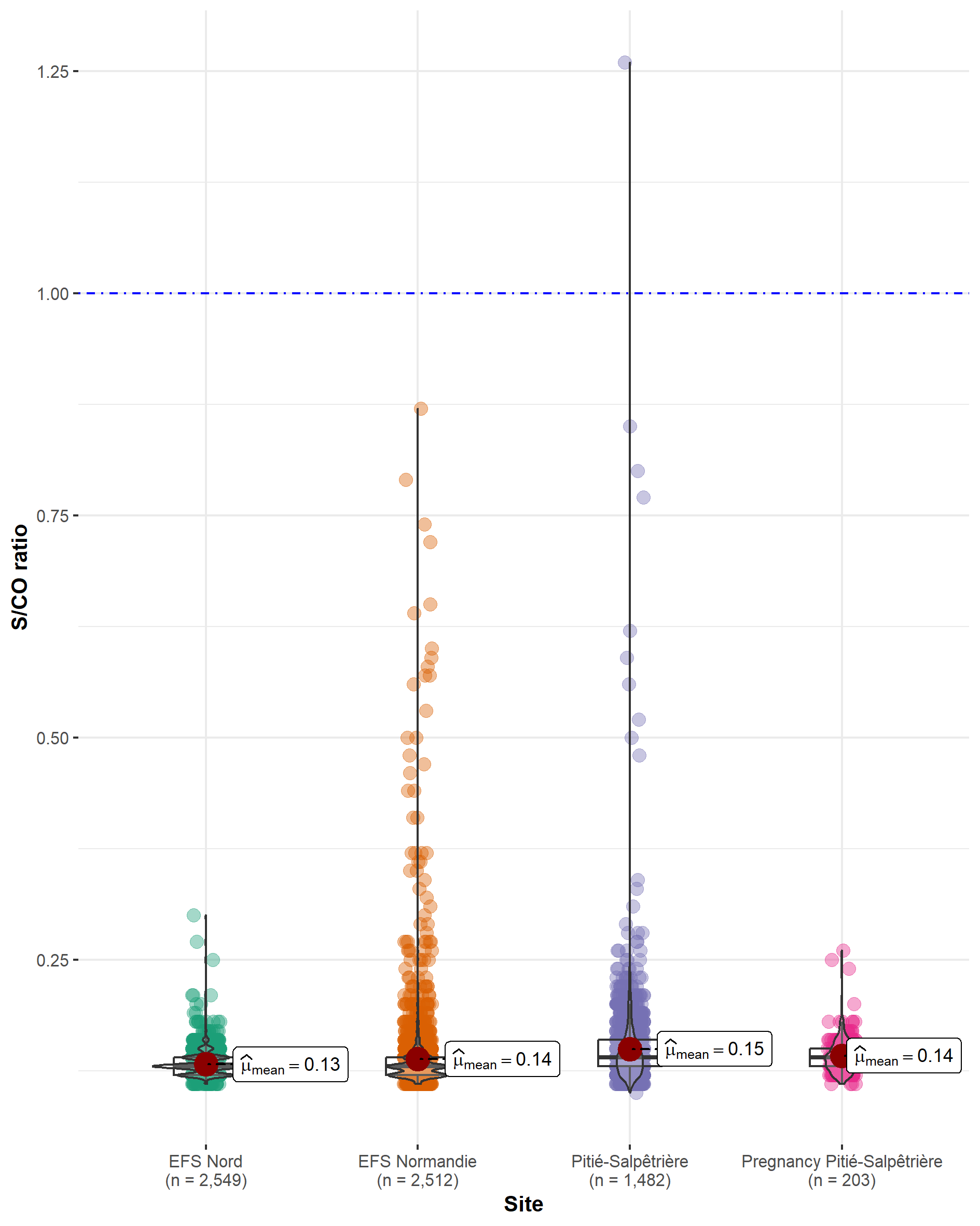

Supplement: Fig S1B — Image of Fig. S1B. [file jcm.00095-24-s0003.tiff]
