# Supplementary material for: Sensitivity and specificity of the new Bio-Rad HIV screening test, Access HIV combo V2
Source: J Clin Microbiol. 2024 Mar 27;62(5):e00095-24. doi: 10.1128/jcm.00095-24 (PMC11077987; doi:10.1128/jcm.00095-24)
Supplement: Figure S1 — S/CO values for HIV-positive samples (A) and HIV-negative samples (B), according to the sites. [file jcm.00095-24-s0004.docx]

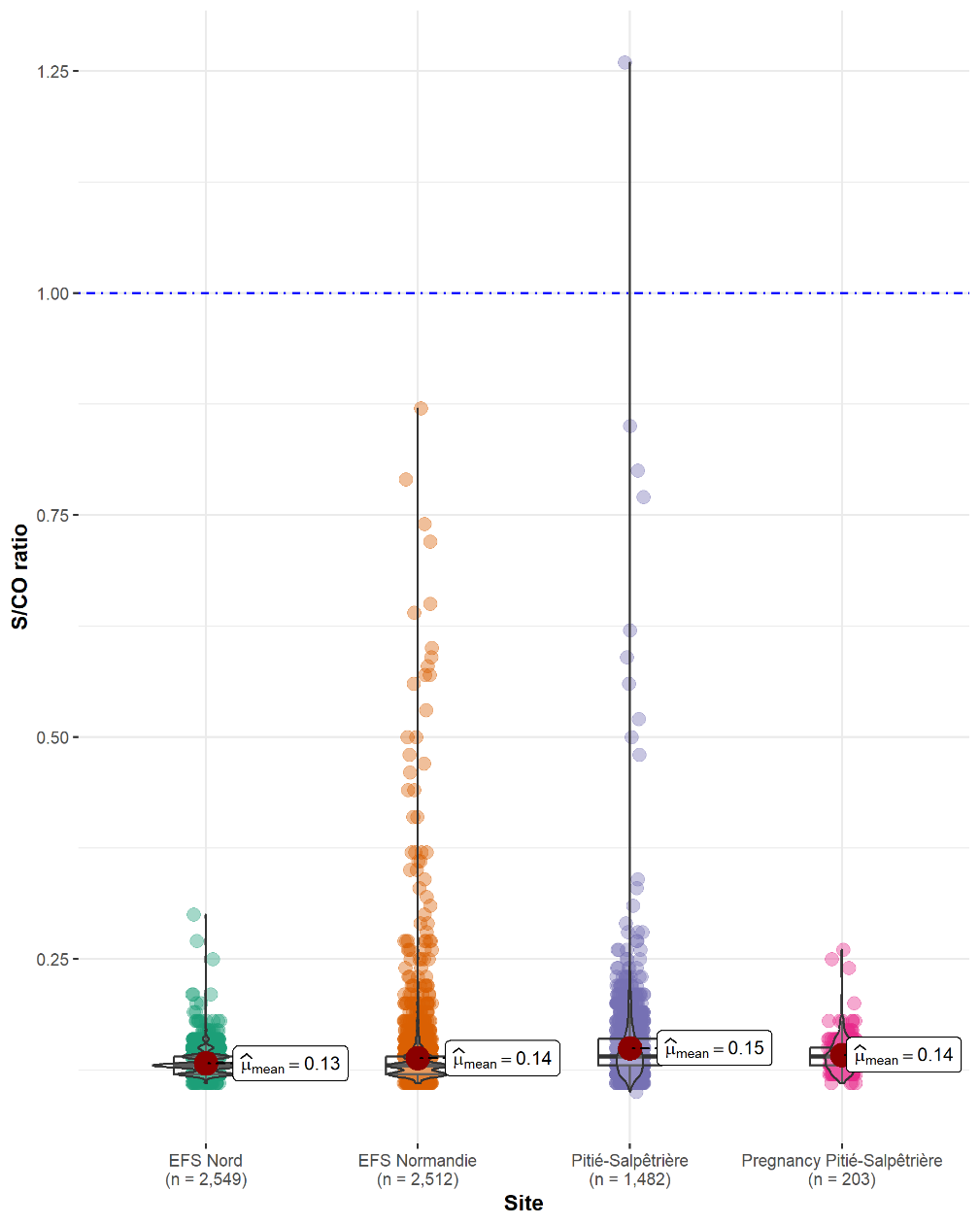

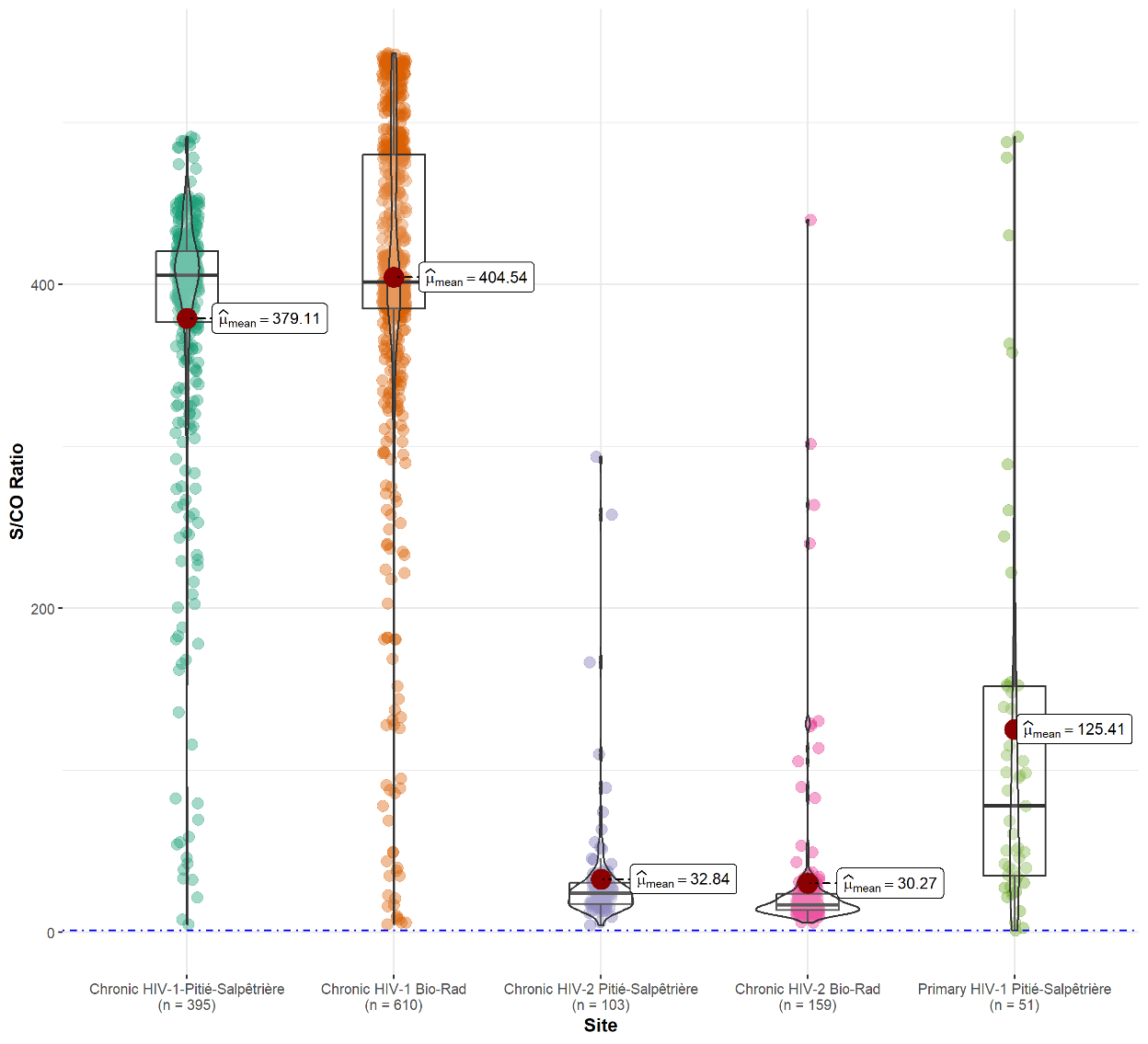


Sup. fig 1: S/CO values for HIV-positives samples (A) and HIV-negatives samples (B), according to the sites. For the HIV-1 positive at Pitié-Salpêtrière Hospital, the 33 samples with reactivity over the quantification limit were excluded from the plot. For repeated samples, only the repeated value is shown. Boxplot represent (bottom to top): first quartile, median and third quartile. Blue dotted line represents S/CO = 1.

**B**

**A**
