# Supplementary material for: Sensitivity and specificity of the new Bio-Rad HIV screening test, Access HIV combo V2
Source: J Clin Microbiol. 2024 Mar 27;62(5):e00095-24. doi: 10.1128/jcm.00095-24 (PMC11077987; doi:10.1128/jcm.00095-24)
Supplement: Supplemental tables — Tables S1 to S3. [file jcm.00095-24-s0005.docx]

Supplementary table 1: Subtypes and recombinant forms of retrospective HIV-1 samples used at Pitié-Salpêtrière Hospital.

| Subtype/ group/CRF | Stage | No Specimens |
| --- | --- | --- |
| Subtype A | Chronic | 34 |
| Subtype B | Chronic | 94 |
| Subtype C | Chronic | 20 |
| Subtype D | Chronic | 14 |
| Subtype F | Chronic | 17 |
| Subtype G | Chronic | 29 |
| Subtype H | Chronic | 6 |
| Subtype J | Chronic | 1 |
| Subtype K | Chronic | 2 |
| Group O | Chronic | 3 |
| CRF01 | Chronic | 22 |
| CRF02 | Chronic | 97 |
| CRF06 | Chronic | 15 |
| CRF08 | Chronic | 1 |
| CRF09 | Chronic | 7 |
| CRF10 | Chronic | 1 |
| CRF11 | Chronic | 8 |
| CRF13 | Chronic | 6 |
| CRF14 | Chronic | 6 |
| CRF15 | Chronic | 2 |
| CRF18 | Chronic | 3 |
| CRF19 | Chronic | 2 |
| CRF20 | Chronic | 1 |
| CRF22 | Chronic | 2 |
| CRF25 | Chronic | 1 |
| CRF30 | Chronic | 1 |
| CRF36 | Chronic | 2 |
| CRF37 | Chronic | 1 |
| CRF42 | Chronic | 1 |
| CRF44 | Chronic | 1 |
| CRF45 | Chronic | 1 |
| CRF60 | Chronic | 2 |
| Subtype A | Primary | 1 |
| Subtype B | Primary | 20 |
| Subtype C | Primary | 2 |
| Subtype D | Primary | 1 |
| CRF01/CRF15^a^ | Primary | 1 |
| CRF02 | Primary | 10 |
| CRF06 | Primary | 4 |
| CRF18 | Primary | 2 |
| Unknown | Primary | 8 |

^a^ Genotyping was unable to distinguish between CRF01 and CRF15

Supplementary table 2: Commercial seroconversion panels used for the present study with results for Access HIV combo V2 and Abbott’s Architect.

| Vendor | Sample ID | Days to first reactive result | | Source for Architect results |
| --- | --- | --- | --- | --- |
|  |  | Access Combo V2 | Architect |  |
| Seracare / BBI | PRB944 | 2 | 7 | FDA notice [1] |
|  | PRB945 | 7 | 13 | T. Sano et al [2] |
|  | PRB949 | 18 | 18 | Manufacturer |
|  | PRB950 | 18 | 18 | Manufacturer |
|  | PRB953 | 7 | 3 | Manufacturer |
|  | PRB954 | 17 | 17 | Manufacturer |
|  | PRB955 | 3 | 3 | Manufacturer |
|  | PRB957 | 16 | 23 | FDA notice [1] |
|  | PRB958 | 7 | 7 | FDA notice [1] |
|  | PRB964 | 22 | 22 | Manufacturer |
|  | PRB966 | 44 | 44 | Manufacturer |
|  | PRB969 | 63 | 63 | Manufacturer |
|  | PRB970 | 0 | 0 | Manufacturer |
|  | PRB973 | 7 | 7 | Manufacturer |
|  | PRB975 | 14 | 14 | Manufacturer |
|  | SC-0600-0270 | 30 | 30 | Manufacturer |
|  | SC-0600-0271 | 7 | 7 | Manufacturer |
|  | SC-0600-0272 | 18 | 18 | Manufacturer |
| Zeptometrix | SC9011 | 36 | 36 | Manufacturer |
|  | SC9012 | 16 | 16 | Manufacturer |
|  | SC9013 | 25 | 25 | Manufacturer |
|  | SC9016 | 30 | 30 | Manufacturer |
|  | SC9018 | 25 | 28 | Manufacturer |
|  | SC9020 | 90 | 90 | Manufacturer |
|  | SC9021 | 47 | 47 | Manufacturer |
|  | SC9023 | 78 | 78 | Manufacturer |
|  | SC9024 | 53 | 53 | Manufacturer |
|  | SC9025 | 85 | 85 | Manufacturer |
|  | SC9026 | 44 | 44 | Manufacturer |
|  | SC9030 | 47 | 47 | Manufacturer |
|  | SC9031 | 146 | 146 | Manufacturer |
|  | SC9033 | 82 | 82 | Manufacturer |
|  | SC9089 | 16 | 16 | Manufacturer |
|  | SC6244 | 28 | 28 | Manufacturer |
|  | SC12008 | 23 | 28 | Manufacturer |
| Biomex | SCP-HIV-002 | 63 | 63 | Manufacturer |
|  | SCP-HIV-003 | 17 | 17 | Manufacturer |
|  | SCP-HIV-004 | 56 | 56 | Manufacturer |
|  | SCP-HIV-005 | 16 | 16 | Manufacturer |
|  | SCP-HIV-006 | 15 | 15 | Manufacturer |
|  | SCP-HIV-007 | 12 | 12 | Manufacturer |

Supplementary table 3: Summary of the p24 antigen limit of detection (IU/mL) on the WHO panel for six 4^th^ generation assays. Data for comparative assays were extracted from Qiu et al. [3].

|  | Access HIV combo V2 | ARCHITECT HIV Ag/Ab Combo | Liaison® XL murex HIV ab/Ag HT | Elecsys HIV Duo | Elecsys® HIV combi PT | BioPlex 2200 HIV Ag-Ab |
| --- | --- | --- | --- | --- | --- | --- |
| Median (IQR)^1,2^ | 0.43  (0.38-0.56) | 0.57  (0.43-0.64) | 0.67  (0.58-0.72) | 0.33  (0.30-0.37) | 0.89  (0.74-1.04) | 0.27  (0.21-0.36) |
| P-value for comparison with Access^3^ | NA^4^ | 0.24 | **0.0012** | **0.02** | **0.0005** | 0.13 |

1: Inter Quartile Range

2: Results are expressed as IU/mL

3: Based on Wilcoxon’s test for paired samples

4: Not applicable

Bibliography for Supplementary tables 2 and 3:

[1] ARCHITECT HIV Ag/Ab Combo package insert, 2010. https://www.fda.gov/media/116836/download.

[2] T. Sano, M. Kondo, Y. Yoshimura, N. Tachikawa, H. Sagara, I. Itoda, K. Yamanaka, K. Sudo, S. Kato, M. Imai, Evaluation of a New Vesion of the Human Immunodeficiency Virus Antigen and Antibody Combination Assay with Improved Sensitivity in HIV-1 p24 Antigen Detection, J. J. A. Inf. D. 87 (2013) 415–423. https://doi.org/10.11150/kansenshogakuzasshi.87.415.

[3] X. Qiu, L. Sokoll, T. Duong Ly, C. Coignard, S.H. Eshleman, P. Mohr, C. Huizenga, P. Swanson, G. Cloherty, J. Hackett Jr., An improved HIV antigen/antibody prototype assay for earlier detection of acute HIV infection, Journal of Clinical Virology. 145 (2021) 105022. https://doi.org/10.1016/j.jcv.2021.105022.
